# Supplementary material for: Splice-Junction-Based Mapping of Alternative Isoforms in the Human Proteome
Source: Cell Rep. Author manuscript; Available in PMC 2020 Jan 15. (PMC6961840; doi:10.1016/j.celrep.2019.11.026)
Supplement: 2 [file NIHMS1546469-supplement-2.zip › DF1/PXD010154/Lung/Lung_43_CNTN6_FFMLLPLINSSAGDGLLSRPIFTQEPHDVIFPLDLSK.pdf]

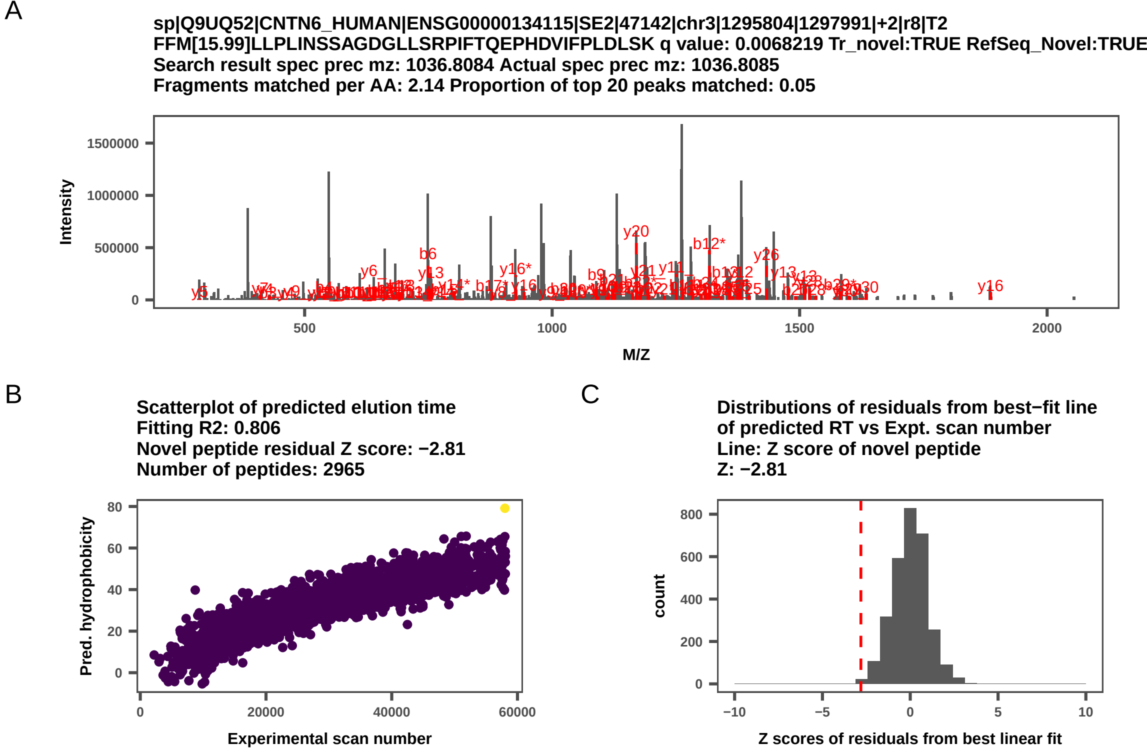
A
sp|Q9UQ52|CNTN6_HUMAN|ENSG00000134115|SE2|47142|chr3|1295804|1297991|+2|r8|T2
FFM[15.99]LLPLINSSAGDGLLSRPIFTQEPHDVIFPLDLSK q value: 0.0068219 Tr_novel:TRUE RefSeq_Novel:TRUE
Search result spec prec mz: 1036.8084 Actual spec prec mz: 1036.8085
Fragments matched per AA: 2.14 Proportion of top 20 peaks matched: 0.05
Intensity
1500000
1000000
500000
0
y20
b12*
b6
y26
y6
y13
y16*
y11
b13
y12
y13
y16
M/Z
500
1000
1500
2000
B
Scatterplot of predicted elution time
Fitting R2: 0.806
Novel peptide residual Z score: −2.81
Number of peptides: 2965
Pred. hydrophobicity
80
60
40
20
0
0
20000
40000
60000
Experimental scan number
C
Distributions of residuals from best−fit line
of predicted RT vs Expt. scan number
Line: Z score of novel peptide
Z: −2.81
count
800
600
400
200
0
−10
−5
0
5
10
Z scores of residuals from best linear fit
